# Alternate to soybean meal: evaluation of guar korma on growth performance, nutrient utilization and blood biochemical indices in water buffalo (*Bubalus bubalis*) calves

**DOI:** 10.3389/fvets.2026.1882967

**Published:** 2026-07-09

**Authors:** Avijit Dey, Pulkit Sheoran, Prerna Thuwal, Anirban Mahato, Rajesh Kumar, Sanjay Kumar, Tirtha Kumar Datta

**Affiliations:** 1 Division of Animal Nutrition and Feed Technology, ICAR-Central Institute for Research on Buffaloes, Hisar, India; 2 Division of Animal Genetics and Breeding, ICAR-Central Institute for Research on Buffaloes, Hisar, India

**Keywords:** animal performance, feeding module, health status, non-conventional feed, voluntary feed intake

## Abstract

**Introduction:**

Soybean meal is a high protein, one of the major feed ingredients used in calf ration. However, high cost and limited availability restricts its application in practical ration formulation. Guar korma is a non-conventional agro-industrial by-product from guar industry, rich in protein. Therefore, this study was conducted to assess the effects of substituting soybean meal with varying proportions of guar korma on the performances of Murrah buffalo calves.

**Methods:**

Twenty-four Murrah buffalo male calves (9–16 months old, average body weight of 206.18 ± 40.67 kg) were randomly allotted to three groups in a completely randomised design and subjected to three iso-nitrogenous dietary treatments consisted of graded levels of roasted guar korma, namely 0% (CON), 25% (GK-25) and 50% (GK-50), replacing soybean meal in the concentrate mixture along with wheat straw and green oats. The roasted guar korma was procured from local market and the feeding trial continued for 120 days, where voluntary feed intake, fortnightly body weight changes, feed digestibility by total collection of faeces and blood biochemical parameters (Hb, PCV, serum biochemistry) were monitored.

**Results:**

The replacement of soybean meal with guar korma up to 50% level did not (*p* > 0.05) affect dry matter intake, growth performance and haematological parameters in buffalo calves. All the animals irrespective of dietary regimes showed comparable (*p* > 0.05) feed efficiency and nutrient utilization. As guar korma is locally available at low cost (INR 38 vs. 55/kg; Guar korma vs. Soybean meal), there was significant reduction (INR 2210/tonne feed) in daily feeding cost (INR 5.42) with increasing guar korma in the concentrate mixture (50% inclusion) in the current production system.

**Discussion:**

The study indicates that roasted guar korma can be used as an affordable alternative protein source to partially replace soybean meal in buffalo calf diets owing to absence of significant differences among treatments. However, future research may be carried out on its effectiveness in influencing milk production and composition with the experimental model investigated in the present study.

## 1 Introduction

Livestock farming plays a vital role in supporting rural livelihoods across Asian countries. India holds a leading position globally, supporting around 13% of the world’s livestock, comprising 57% of buffaloes and 16% of cattle (1). According to the 20th Livestock Census of India (2), the nation has a buffalo population of about 110 million heads. Among the recognized breeds, Murrah buffaloes (42% of the total buffalo population of the nation), which originated in the Haryana region and are now widely distributed across the country, are valued for their high milk yield and adaptability to diverse production systems. Under semi-intensive management practiced by smallholder farmers, Murrah buffaloes are noted for producing milk with high fat content (6.5–9.0%), exhibiting good resistance to endemic diseases, and tolerating tropical climatic conditions. However, a large proportion of buffaloes reared by small-scale farmers depend mainly on grazing and crop residues, which often fail to fulfil their complete nutritional requirements (3).

Progressive shrinkage of grazing resources, coupled with increasing demand and rising costs of conventional concentrate feed ingredients, along with an acute shortage of protein supplements for ruminants in developing countries, has necessitated the adoption of non-conventional feed resources such as crop residues and agro-industrial by-products in livestock nutrition (4). Incorporating unconventional feed resources as alternatives to conventional feeds can contribute to reduced feed and production costs (5). Conventional protein supplements such as soybean meal possess a well-balanced amino acid profile and exhibit high ruminal degradability, thereby supporting efficient growth in livestock (6). However, owing to its widespread use and relatively high cost in animal diets, considerable efforts have been directed toward identifying alternative protein-rich non-conventional feed resources for partial or complete replacement.

Guar korma is a potential source of protein for animal feed formulation, derived as a by-product from guar gum processing industry. The original agricultural crop, guar (*Cyamopsis tetragonoloba*) is an important cash crop in rain-fed regions, particularly in the semi-arid and desert areas of India and other Asian countries (7). India alone accounts for approximately 90% of the world’s guar production (8). The Rajasthan province of the country alone contributes approximately 80% of the total guar production, with the remaining share coming from Haryana, Gujarat, Punjab, Uttar Pradesh, and Madhya Pradesh province (9). Guar gum is an economically important product, primarily exported to the United States and European countries for use in cosmetics and various industrial applications. Guar meal, a non-conventional by-product of the guar gum industry, serves as a valuable protein-rich feed for livestock. During processing, the endosperm of the guar seed is utilized for gum extraction, while the remaining germ and hull, obtained in an approximate ratio of 1:3, form guar meal (10). The powdered form of guar meal, known as guar *churi*, contains a comparatively lower protein content (35–40%). In contrast, the granular form, called guar *korma*, is a richer source of crude protein (45–55%) and is characterized by a high proportion of true protein. It also contains significantly higher arginine levels, nearly double those found in soybean meal, making it a promising alternative protein source for high-producing livestock and poultry (11). Although, the industrial guar meal contains some anti-nutritional factors such as trypsin inhibitor, saponins, phytate and tannins but roasting reduces their concentrations significantly, making guar korma a powerhouse of protein source (12).

Guar korma is generally recommended at 5–10% in the concentrate mixture of ruminants. An investigation on replacement of sunflower meal by guar korma suggested 10% level of inclusions in the concentrate mixture of Egyptian buffaloes (13). Shweta et al. (14) reported that 75% inclusion of guar meal by replacing soybean meal in the diet of Nagavali ram lambs enhanced growth rate and protein digestibility. However, a reduction in growth and feed utilization was demonstrated in Barki lambs fed total mixed ration with 10% non-roasted guar meal (15). Experiments on replacement of groundnut cake with 50% toasted guar meal revealed comparative growth rate in Mahabubnagar local kids, however, a reduced growth was reported with 100% substitution (16). Sharif et al. (17) described no variation in growth and nutrient utilization of Sahiwal calves on replacement of cottonseed cake with guar meal.

Buffaloes are an integral part of the livestock production system and play a significant role in food security and poverty alleviation in Asian countries. With more than 90% of the global buffalo population present in Asia, 77.9% buffaloes are inhabitants of South Asian countries. India is home for 57% world’s buffalo population and contributes nearly 50% of the country’s total milk production. Therefore, feed formulation with conventional protein sources to enhance production performances with lowered feed cost remained challenging. Guar korma is rich in protein and locally available at low cost (INR 38 vs. 55/kg; Guar korma vs. Soybean meal). Therefore, economically affordable guar korma may contribute to improvement in overall farm profitability to the resource-constrained small and marginal farmers of the region. Despite its potential, in-depth information on the nutritional value of guar korma, as well as its level of inclusions, effects on growth performance, feed efficiency, and overall economic benefits in livestock especially in buffaloes, remains restricted. Therefore, the present study was undertaken to evaluate the effects of replacing soybean meal with graded levels of roasted guar korma on growth performance, feed efficiency, nutrient utilization and haemato-biochemical parameters in buffalo calves, while also assessing the economic feasibility of this substitution.

## 2 Materials and methods

### 2.1 Ethical statement and site of experiment

The experiment was conducted in the Animal Nutrition and Feed Technology Division of the ICAR-Central Institute for Research on Buffaloes, Hisar, India (29.1203 N, 75.8069 E). All the study protocols applied for animal care, welfare, management, experimental procedure, and sampling were approved by the Institutional Animal Ethics Committee (IAEC) for the care of the buffalo calves and the procedures used in experimentation (IAEC/CIRB/1/2022 dated July 4, 2022).

### 2.2 Experimental animals, management, feeding, and layout

**Figure 1:**
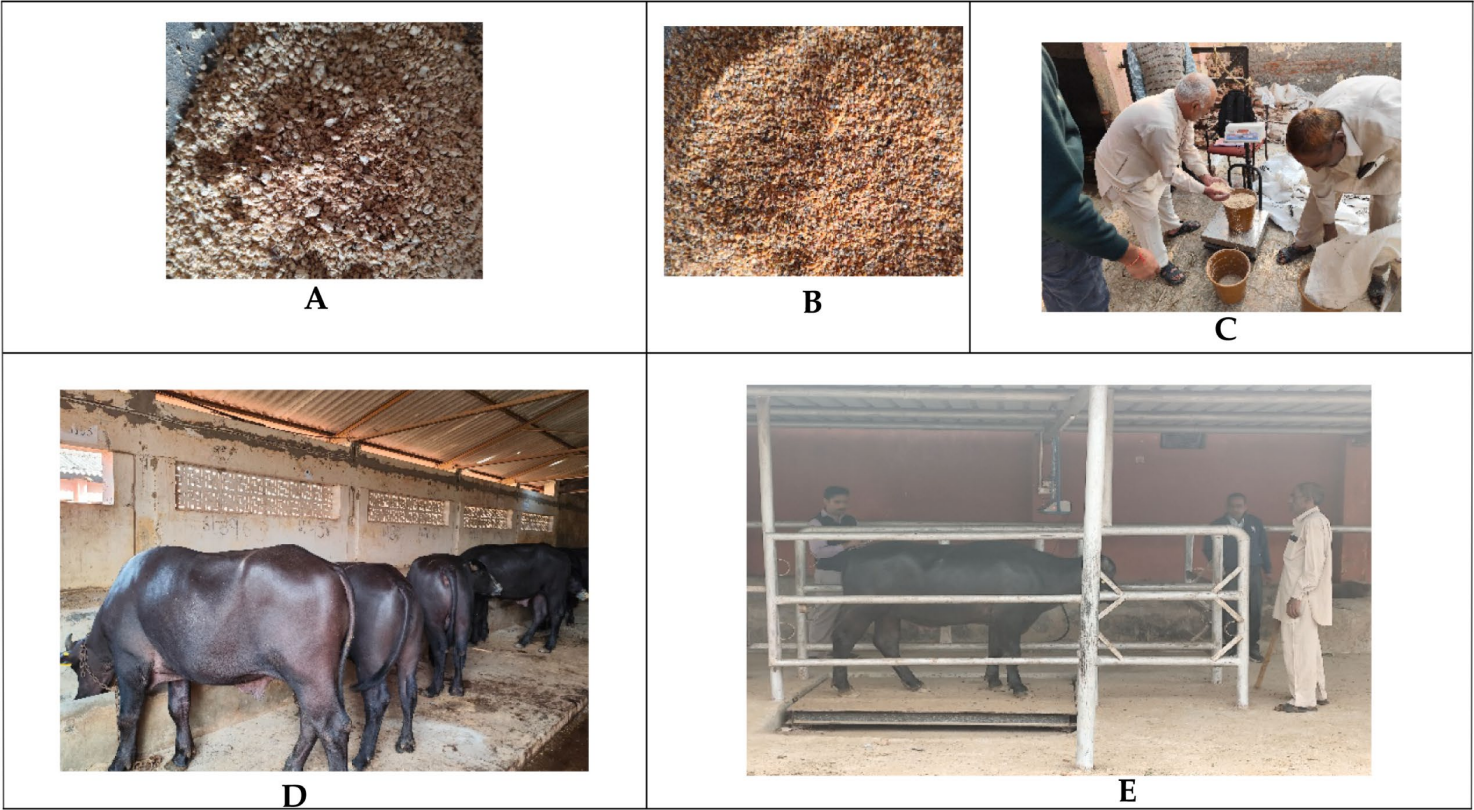
Feeding of guar korma to experimental Murrah buffalo calves. **(A)** Soybean meal; **(B)** guar korma; **(C)** weighing of concentrate mixture with guar korma; **(D)** experimental buffalo calves; **(E)** body weight measurement of buffalo calves.

Twenty-four Murrah buffalo male calves (9–16 months old, avg. body weight of 206.18 ± 40.67 kg) were randomly allocated into three homogeneous groups of eight animals each, based on comparable mean body weight. The animals were distributed among the three groups in such a way that the average initial age and body weight of the groups was comparable, i.e., 1 year 29 days, 205.28 kg (CONT); 1 year 1 month 17 days, 207.15 kg (GK-25), and 1 year 1 month 9 days, 206.09 kg (GK-50). The feeding trial was conducted for a period of 135 days, which included 15 days adaptation and 120 days data recordings, completed during December 2024 (Figure 1). The calves were offered *ad libitum* wheat straw and 5 kg green oats (*Avena sativa*) as roughage sources and formulated concentrate mixtures to the respective group of calves to meet the nutrient requirement (ICAR, 2013). Routine deworming and vaccination were carried out following standard management practices. Details of the chemical composition of feeds and dietary treatments assigned to each experimental group are presented in Table 1.

**Table 1:** Ingredients and chemical compositions of concentrate mixtures, soybean meal, guar korma, wheat straw and oats green fed to buffalo calves.

Parameters	Concentrate mixture-1 (CONT)	Concentrate mixture-2 (GK-25)	Concentrate mixture-3 (GK-50)	Soybean meal	Guar korma	Wheat straw	Oats green
Ingredients (%)							
Wheat grain	30.00	30.00	30.00	**–**	**–**	**–**	**–**
Oats grain	6.00	6.00	6.00	**–**	**–**	**–**	**–**
Wheat bran	35.00	35.00	35.00	**–**	**–**	**–**	**–**
Soybean meal	26.00	19.50	13.00	**–**	**–**	**–**	**–**
Guar korma	–	6.50	13.00	**–**	**–**	**–**	**–**
Mineral mixture	2.00	2.00	2.00	**–**	**–**	**–**	**–**
Common salt	1.00	1.00	1.00	**–**	**–**	**–**	**–**
Chemical composition (%DM)							
Organic matter	90.09	88.79	86.56	93.97	94.05	89.07	89.29
Crude protein	20.64	20.53	20.36	46.48	46.27	4.26	10.03
Ether extract	3.08	3.55	4.12	3.07	5.01	2.03	2.20
Total ash	9.91	11.21	13.44	6.03	5.95	10.93	10.71
Neutral detergent fibre	34.56	36.30	39.14	20.71	22.30	76.47	56.76
Acid detergent fibre	7.62	9.61	10.80	14.33	12.43	47.93	31.35

Guar korma was generally used in the buffalo calf ration at 10% level by replacing soybean meal in the concentrate mixture as per normal farm practices of the institute, where no deleterious effects on growth and health attributes were recorded. As guar korma is available at lower cost than soybean meal, it was thought to include more quantity in the concentrate mixture to make it cost effective. In the present study, the institute provided the roasted guar korma, produced in Haryana province of India. The basal concentrate mixture (CONT) comprised of wheat (30%), oats (6%), deoiled soybean meal (26%), wheat bran (35%), mineral mixture (2%), and common salt (1%). Based on preliminary *in vitro* study at authors’ laboratory, in the experimental diets, deoiled soybean meal as the primary source of crude protein in the concentrate mixture, was partially replaced with the guar korma at 25% (GK-25) and 50% levels (GK-50) in treatment groups, respectively (Table 1).

### 2.3 Feed intake, body weight gain and feed efficiency

A measured quantity of green fodder, wheat straw, and concentrate mixture was offered daily to each animal. Dry matter intake and nutrient intake for individual buffalo calf was calculated based on voluntary feed consumption, considering the average daily feed offered and measurement of refusals during three consecutive days individual feeding at every fortnight. Initial and final body weights of all the calves in each treatment group were recorded using a digital calibrated weighing balance. Body weights were measured early in the morning (08:00 a.m.) before offering feed and water on two consecutive days at the beginning of the experiment and subsequently at fortnightly intervals throughout the experimental period to monitor weight changes.

Average daily body weight gain was calculated accordingly. Feed conversion ratio (FCR) and percent feed efficiency (FE) were computed based on body weight gain and dry matter intake, as presented in Table 2.

**Table 2:** Feed intake, body weight gain and feed efficiency of buffalo calves fed different dietary regimens containing soybean meal and guar korma.

Attributes	Treatments			SEM	*p* Value
	CONT	GK-25	GK-50		
Body weight (kg)					
Initial	205.28	207.15	206.09	10.37	0.997
Final	319.88	317.50	318.25	9.98	0.996
Total gain^a^	114.60	110.35	112.16	1.74	0.625
ADG (g)	954.87	919.49	934.66	14.46	0.626
Dry matter intake (kg/d)					
Wheat straw	1.99	2.05	2.12	0.090	0.858
Conc. mix	2.69	2.69	2.69	0	1.00
Oats green fodder	0.992	0.992	0.992	0	1.00
Total	5.67	5.72	5.79	0.090	0.856
FCR	5.94	6.28	6.23	0.135	0.570
FE (%)	16.95	16.12	16.23	0.338	0.573

a After 120 days.

ADG, average daily gain; FCR, feed conversion ratio (kg DMI/kg gain); FE (%) = Percent Feed Efficiency (kg gain*100/kg DMI).

### 2.4 Measurement of nutrient utilization

To evaluate nutrient utilization efficiency and total tract apparent digestibility, a digestion trial of 8 days duration was conducted consisting of initial 2 days discard of faeces and next 6 days total collection of faeces from each animal. Daily samples of feed offered, feed refusals, and faeces were collected from each calf. Therefore, six representative samples were collected from each animal during the digestion trial. The collected samples were dried daily and mixed for individual calf. After 6 days collection, the samples were ground and analysed for proximate composition by AOAC methods (18) and fibre fractions by van Soest procedures (19). Based on nutrient intake and faecal nutrient output, apparent digestibility coefficients were calculated. The nutrient intake and digestibility percentages of buffalo calves fed different dietary treatments are presented in Tables 3, 4.

**Table 3:** Nutrient digestibility (%) of buffalo calves fed different dietary regime.

Attributes	Treatments			SEM	*p* Value
	CONT	GK-25	GK-50		
Dry matter	62.09	63.07	62.99	0.506	0.698
Organic matter	63.93	64.62	63.45	0.464	0.607
Crude protein	63.51	61.56	65.60	1.011	0.274
Ether extract	74.22^a^	77.87^ab^	79.08^b^	0.767	0.019
Neutral detergent fibre	52.52	54.54	54.62	0.811	0.506
Acid detergent fibre	44.69	47.16	48.22	0.123	0.152

^a,b^Mean values with different superscripts within a row differ significantly (*p* < 0.05).

**Table 4:** Nutritive value and plane of nutrition of buffalo calves fed graded levels of guar korma.

Attributes	Treatments			SEM	*p* value
	CONT	GK-25	GK-50		
Body weight (kg)	311.38	308.38	312.63	10.07	0.986
Metabolic size (kg W^0.75^)	73.93	73.41	74.22	1.79	0.984
Nutrient intake					
*Digestible DM*					
g kg^−1^ BW	11.54	11.95	11.97	0.402	0.895
g kg^−1^ W^0.75^	48.07	49.70	49.94	1.38	0.846
*Digestible OM*					
g kg^−1^ BW	10.69	10.95	10.65	0.361	0.941
g kg^−1^ W^0.75^	44.56	45.55	49.94	1.23	0.931
*Digestible CP*					
g kg^−1^ BW	1.56	1.51	1.57	0.057	0.893
g kg^−1^ W^0.75^	6.48	6.26	6.56	0.190	0.811
^a^ *TDN*					
g kg^−1^ BW	11.22	11.50	11.19	0.379	0.942
g kg^−1^ W^0.75^	46.79	47.82	46.69	1.29	0.930
*Nutrient density (%)*					
DCP	13.09	12.92	12.76	0.135	0.630
TDN	60.45	60.67	58.88	0.457	0.226

a TDN calculated from digestible OM (1 kg digestible OM = 1.05 kg TDN; 37).

DM, dry matter; OM, organic matter; CP, crude protein; TDN, total digestible nutrients.

### 2.5 Hemato-biochemical parameters

Blood samples were collected from the jugular vein of each animal at the start of the experiment (day 0) and at the end of the feeding trial (120 days), in the morning prior to the provision of feed and water. Approximately 10 mL of blood was collected in vacutainers without anticoagulant. Serum was separated after allowing the blood to clot followed by centrifugation and was stored at −20 °C until further analysis. Serum concentrations of urea, haemoglobin, packed cell volume (PCV), glucose, total protein, albumin, alanine aminotransferase (ALT), aspartate aminotransferase (AST), and alkaline phosphatase (ALP) were determined using commercial diagnostic kits (Coral Clinical Systems, India) with an automated biochemical analyser (Erba, Transasia).

### 2.6 Statistical analysis

All data pertaining to the measured parameters were analysed using a completely randomized design through the general linear model procedure of IBM SPSS, version 30.0.0 (20), considering dietary treatment as a fixed effect. During the animal experimentation, each animal within a treatment formed an experimental unit. The statistical model *Yij* = *μ* + *Ti* + *ɛij* was used, where *Yij* = individual observation, *μ* = overall mean, *Ti* = effect of *i*th treatment, and *ɛij* = residual error. Differences among treatment means were evaluated using one-way analysis of variance (ANOVA) as described by Snedecor and Cochran (21). Repeated measures ANOVA were used for analysis of body weight changes over the time period. Mean comparisons were further performed using Tukey’s HSD test, and statistical significance was declared at *p* ≤ 0.05.

## 3 Results

### 3.1 Chemical composition of diets

The feed ingredients and chemical composition of experimental diets fed to Murrah buffalo calves are presented in Table 1. All the three concentrate mixtures were iso-nitrogenous, however, they differed (*p* < 0.05) in organic matter, ether extract as well as fibre contents owing to replacement of soybean meal with guar korma. Both soybean meal and guar korma are comparable (*p* > 0.05) in crude protein content, however, they differed in concentrations of other chemical constituents, which ultimately varied the chemical composition of concentrate mixtures.

### 3.2 Voluntary feed intake

The dry matter intake from various feed constituents in buffalo calves under different dietary regimes is presented in Table 2. Since, the total dry matter intake (kg/day) was marginally higher in buffaloes under the GK-50 group compared to the other groups, the difference was not statistically significant (*p* > 0.05). The intake of wheat straw showed minor variations among treatments; however, these differences were also non-significant (*p* > 0.05). Intake of concentrate mixture and green oats fodder remained comparable across all treatments, reflecting uniform consumption of these feed components.

### 3.3 Growth performance and feed efficiency

**Figure 2:**
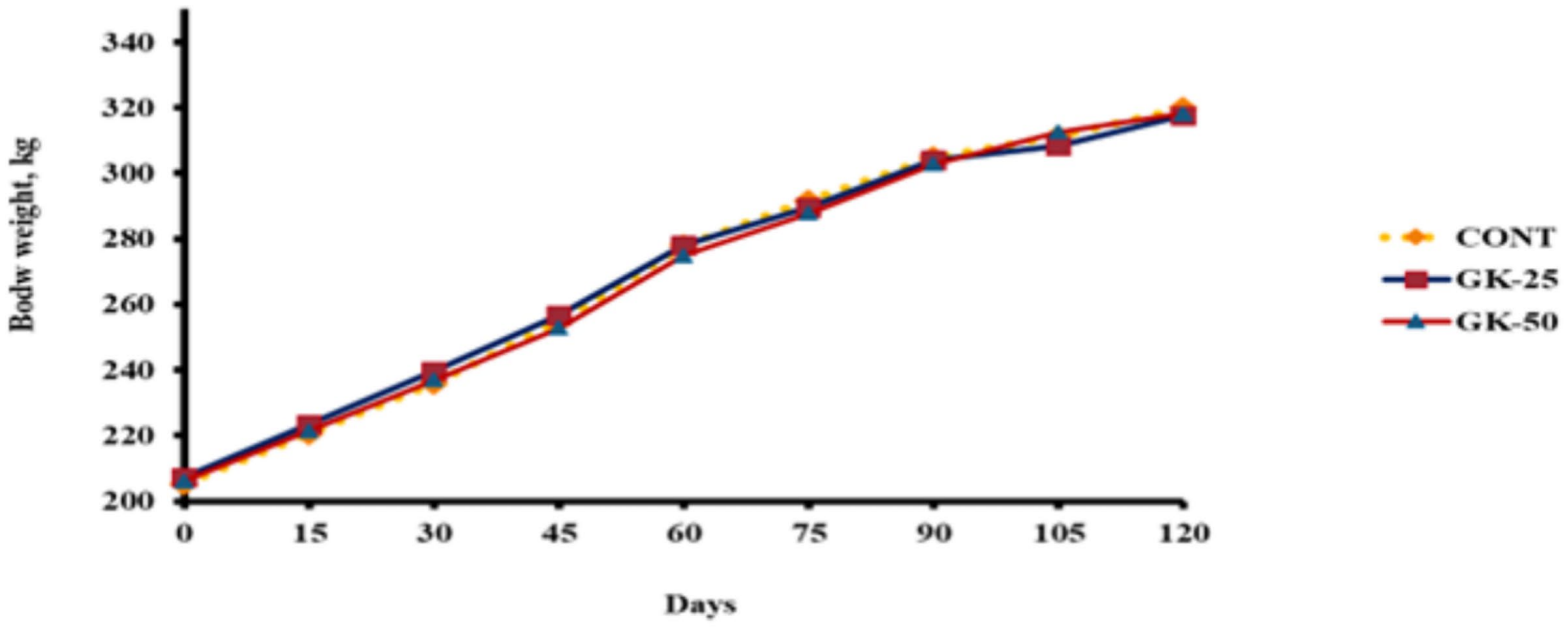
Fortnightly body weight changes of buffalo calves fed different dietary regimes.

Growth performance parameters of buffalo calves under different dietary treatments are presented in Table 2. Initial and final body weights did not differ (*p* > 0.05) among the control and treatment groups, indicating that the type of diet had no measurable effect on baseline or final body weights. Similarly, total body weight gain and average daily gain (ADG) were comparable (*p* > 0.05) across treatments, demonstrating that all dietary regimens supported similar growth performance over the 120-day experimental period (Figures 1, 2).

Feed conversion ratio (FCR) and percent feed efficiency (FE%) were not significantly affected by the dietary treatments (*p* = 0.570 and 0.573, respectively). This findings indicate that replacing soybean meal with guar korma in the concentrate mixture did not adversely affect feed utilization efficiency (Table 2).

### 3.4 Nutrient digestibility and utilization

The apparent digestibility coefficients (Table 3) of dry matter, organic matter, crude protein, neutral detergent fibre, and acid detergent fibre did not vary significantly (*p* > 0.05) among the three dietary treatments. In contrast, ether extract digestibility differed among groups, with the highest value observed in the GK-50 group (*p* = 0.019), followed by the GK-25 group, compared with the control (CONT).

Body weight and metabolic size (kg W^0.75) of buffalo calves were comparable (*p* > 0.05) across the control and guar korma–supplemented groups (GK-25 and GK-50). Likewise, nutrient intake expressed as digestible dry matter, organic matter, crude protein, and total digestible nutrients per kilogram of body weight as well as per kilogram of metabolic size did not differ significantly (*p* > 0.05) among the treatments (Table 3). Furthermore, the nutrient density of the ration, in terms of percentage of digestible crude protein and total digestible nutrients, remained comparable (*p* > 0.05) across all dietary regimens.

### 3.5 Haematological-biochemical parameters

The evaluated haemato-biochemical parameters included haemoglobin (Hb), packed cell volume (PCV), glucose, total protein, albumin, globulin, albumin–globulin ratio, liver enzymes (AST, ALT, and ALP), and urea (Table 5). All blood parameters remained within the normal physiological range for buffalo calves and did not differ significantly (*p* > 0.05) among the dietary treatments. This indicates that partial replacement of soybean meal with guar korma at different inclusion levels had no detrimental effects on the physiological health status of the buffalo calves throughout the experimental period.

**Table 5:** Haemato-biochemical parameters of buffalo calves supplemented with diet containing graded levels of guar korma.

Parameters	Treatments			SEM	*p* Value
	CONT	GK-25	GK-50		
Hb (g/dL)	10.95	10.98	10.83	0.40	0.989
PCV (%)	35.70	34.43	34.08	1.27	0.882
Glucose (mg/dL)	68.58	66.08	72.80	2.104	0.46
Total protein (g/dL)	6.45	6.10	5.98	0.10	0.124
Albumin (g/dL)	2.34	2.25	2.43	0.06	0.467
Globulin (g/dL)	4.12	3.86	3.54	0.11	0.082
A:G ratio	0.57	0.59	0.69	0.03	0.142
AST (U/L)	149.53	133.98	139.30	3.33	0.153
ALT (U/L)	59.05	55.43	54.38	1.24	0.297
ALP (U/L)	147.75	150.25	146.75	8.67	0.988
Urea (mg/dL)	34.33	37.13	35.28	1.13	0.634

Hb, haemoglobin; PCV, packed cell volume; A:G, albumin-globulin ratio; AST, aspartate aminotransferase; ALT, alanine aminotransferase; ALP, alkaline phosphatase.

### 3.6 Economic efficiency

The prices of raw materials (INR/tonne) used in concentrate mixture preparation were 25,000, 22,000, 23,000, 55,000, 38,000, 10,000 and 5,000 for wheat, oats, wheat bran, soybean meal, guar korma, mineral mixture and common salt, respectively. Consequently, the cost (INR/tonne) of the concentrate mixtures was calculated as follows: CM-1 (33220/−), CM-2 (32110/−) and CM-3 (31010/−). Increasing the guar korma inclusion level in the concentrate mixture resulted lowered cost of concentrate feeds in the ration. Considering current market price of wheat straw (INR 4000/tonne DM) and green oats (INR 30000/tonne DM), the daily feed cost (INR) remained 127.08, 124.34 and 121.66 for the animals under CONT, GK-25 and GK-50, respectively. Therefore, there was a reduction in daily feeding cost of INR 5.42 by 50% replacement of soybean meal with guar korma in the concentrate mixture of buffalo calves.

## 4 Discussion

The chemical composition of the experimental concentrate mixtures revealed comparable (*p* > 0.05) crude protein content across all dietary treatments, indicating that the replacement of soybean meal with guar korma was nutritionally balanced and served as an effective protein substitute for soybean meal without altering the overall nitrogen supply of the diet. This indicates that the formulation strategy successfully maintained isonitrogenous conditions, allowing any observed animal responses to be attributed more to ingredient characteristics than to differences in protein levels.

The slightly higher ether extract content in the GK-50 diet indicates a marginal increase in dietary energy density, which could have contributed positively to energy balance without exceeding levels that might impair rumen fermentation. The findings are in agreement with the study done by Shweta et al. (14) in sheep which suggests that guar korma may not only serve as a protein source but also provide supplementary energy through its inherent oil fraction, potentially improving feed efficiency under certain conditions.

The observed increase in NDF and ADF with higher guar korma inclusion reflects its fibrous composition, however, since intake and digestibility were not adversely affected, it can be inferred that the fiber remained partially fermentable. This balance between structural fiber and digestibility may support optimal rumen fermentation, ensuring efficient nutrient utilization.

Dry matter intake and individual feed component intake did not differ significantly among treatments, indicating good palatability and acceptability of guar korma-based diets by buffalo calves. Typically, inclusion of unconventional protein sources such as guar korma raises concerns regarding residual anti-nutritional factors, particularly gums (galactomannans) and saponins, which can impair intake through reduced palatability or altered rumen function. However, lack of any decline in DMI even at 50% replacement indicates that either the processing of guar korma effectively reduced these compounds or that the inclusion level remained below the threshold that would elicit negative sensory or metabolic responses. The agreement of the present findings with earlier reports in kids (16) and crossbred cattle calves (17, 22, 23) further reinforces the consistency of guar meal’s feeding value across species and dietary contexts. Notably, the ability to replace conventional oilseed cakes (groundnut cake/cottonseed cake) up to 75–100% without compromising intake highlights its potential to reduce dependence on more expensive or regionally scarce protein supplements. This is particularly relevant under conditions where feed cost optimization is critical. Overall, the results suggest that guar korma, when properly processed and incorporated, does not impose palatability constraints or intake-limiting effects, even at relatively high inclusion levels.

The comparable growth rates observed across treatments suggests that the protein fraction from guar korma was not only adequately consumed but also efficiently digested, absorbed, and utilized for tissue accretion, at par with soybean meal. This equivalence is particularly noteworthy given that soybean meal is often considered a benchmark protein source in ruminant nutrition due to its balanced amino acid profile and high digestibility. The present findings indicate that guar korma can serve as a nutritionally competitive alternative, at least under the conditions of this study. The reported similarity in essential amino acid index with soybean meal (24) further suggests that the absorbed amino acid profile may have met the requirements for growth without creating limiting deficiencies. Additionally, the comparable *in vitro* ruminal protein degradability (81.87% vs. 79.72%) values (25) imply that both protein sources likely provided similar proportions of rumen degradable protein (RDP) and rumen undegradable protein (RUP), thereby maintaining an optimal balance between microbial nitrogen supply and bypass protein availability.

The lack of improvement in growth performance, despite adequate protein quality, may reflect a scenario where protein was not the first limiting nutrient in the basal diet. The variability observed across studies—where some report improved growth with guar meal inclusion (26–28) while others (16, 17), including the present study, show no change—can be attributed to several interacting factors. Species differences play a critical role, as small ruminants like sheep and goats may exhibit different digestive kinetics, selective feeding behavior, and tolerance to residual anti-nutritional factors (28, 29) compared to buffalo calves.

The unchanged feed conversion ratio and feed efficiency further reinforce that nutrient utilization efficiency was maintained across treatments. This indicates that metabolic processes governing digestion, absorption, and tissue deposition operated with similar efficiency regardless of protein source. Similar findings were reported by Yadav et al. (30) in buffalo calves on replacing groundnut cake with roasted guar meal. This implies that replacing soybean meal with guar korma did not impair the conversion of feed nutrients into body mass. The similarity in the apparent digestibility of dry matter, organic matter, crude protein, NDF, and ADF across treatments indicates that inclusion of guar korma did not impose any constraints on rumen microbial activity or digestive kinetics. This finding is particularly important because alternative protein sources often affect fibre degradation either through shifts in rumen microbial populations or through the presence of anti-nutritional factors. In the present study, the comparable digestibility coefficients suggest that guar korma supported a stable rumen environment, enabling efficient breakdown of both structural and non-structural carbohydrates.

The ruminal pH is not affected by the level or source of protein (31) and the ruminal degradability (81.87%) of guar korma was also suitable to maintain ruminal ammonia-N concentration, which supported enough ruminal fermentation for digestion of composite diet (25). The results agreed with the earlier research (30), where groundnut cake replacement with roasted guar meal in buffalo calves reported similar nutrient digestibility. However, as reported by Shweta et al. (28), crude protein (CP) digestibility improved significantly (*p* < 0.05) with guar meal inclusion up to 75% in Nagavali ram Lambs. The study by Shwerab et al. (15), where inclusion of guar korma in total mixed ration revealed comparable ruminal pH, volatile fatty acids or microbial protein production in sheep, corroborated our findings in comparable digestibility owing to similar ruminal activities. Species-specific differences in digestive physiology and feeding behavior may also contribute to variability in outcomes, particularly when comparing small ruminants with buffalo calves.

Ether extract digestibility was significantly higher in the GK-50 group compared to the control, which may be attributed to the higher fat content of guar korma. Improved fat digestibility could be due to better emulsification and absorption of dietary lipids, contributing marginally to energy availability. This improvement did not adversely affect fibre digestibility, indicating that the fat level remained below the threshold known to inhibit cellulolytic bacteria or interfere with rumen fermentation. Nutrient utilization parameters expressed per unit body weight and metabolic size did not show any significant differences among dietary treatments. Digestible DM, OM, CP, and TDN intake were comparable across the treatment groups, indicating that the plane of nutrition was maintained irrespective of the protein source and its inclusion level. This suggests that the physiological processes governing digestion, absorption, and assimilation of nutrients were not affected by the nature or inclusion level of the protein source. Such uniformity points toward a well-balanced dietary formulation, where substitution with guar korma did not impose additional metabolic constraints or inefficiencies. The comparable intake of digestible DM, OM, CP, and TDN across treatments implies that the animals regulated their intake or digestion in a manner that ensured consistent availability of utilizable nutrients, regardless of dietary variation. This also suggests that guar korma did not negatively affect palatability or rumen function, both of which could otherwise alter feed intake and nutrient availability. Nutrient density in terms of DCP and TDN also remained unaffected, suggesting that guar korma inclusion did not dilute dietary energy or protein concentration (29, 30), which is critical for maintaining optimal microbial activity and nutrient synchronization in the digestive system. In ruminant systems, even subtle imbalances in protein and energy supply can disrupt microbial efficiency and reduce overall nutrient utilization. The present findings imply that guar korma was effectively integrated into the diet without disturbing this balance.

The haemato-biochemical parameters remained within normal physiological ranges (32, 33) and did not differ significantly among treatments, reflecting an absence of nutritional or metabolic stress. Comparable haemoglobin and PCV values indicated that oxygen-carrying capacity and overall haematological status were not affected by guar korma inclusion. Similarly, stable serum glucose, protein fractions, liver enzymes (AST, ALT, ALP), and urea levels suggest that guar korma did not exert any adverse effects on energy metabolism, protein metabolism, liver function and kidney function (34). Although the blood parameters were analysed at the beginning and end of the study, intermediate measurement could have provided additional information regarding the metabolic adaptation of animals to increasing inclusion levels of guar korma. In ruminants, blood glucose is tightly controlled and affected by gluconeogenesis; thus, unchanged glucose levels indicate that guar korma inclusion did not impair ruminal fermentation patterns or hepatic glucose synthesis. The stability of albumin and globulin across the treatments implies that protein digestion, absorption, and subsequent metabolic utilization were not compromised, and that no inflammatory or immunological challenge was induced by the dietary treatments. The findings are consistent with those of other researchers (28), where the impact of substituting guar korma with soybean meal on the blood and serum profile of Nagavali ram lambs revealed no significant variations.

In the present study, the increase in guar korma (50%) in the ration was demonstrated to reduce the total feed cost (INR 2210/tonne feed) of the ration with daily reduction of feeding cost of INR 5.42 per head. The experimentations on replacement of soybean meal with guar korma in the diet of ruminants, earlier researchers (30, 35), demonstrated that there was a reduction in feeding cost when guar korma was added in the diet. The observed reduction in feed cost with increasing inclusion of guar korma highlights its economic advantage as an alternative protein source, particularly in production systems where feed accounts for the largest share of total input costs. Since conventional protein ingredients like soybean meal are often subject to price volatility and high market demand, partial or complete substitution with a relatively less expensive by-product such as guar korma can substantially improve the cost efficiency of ration formulation (36).

## 5 Conclusion

The study showed that replacing 50% of soybean meal with the non-conventional protein source guar korma had no adverse effect on growth rate, feed digestibility, or nutrient utilization efficiency. This indicates that guar korma can be used as an economical alternative protein source to partially replace soybean meal in buffalo calf diets owing to absence of any detectable negative effects under the present experimental conditions. Since guar korma, a by-product of the guar industry, is abundantly available in the region, its incorporation into feed formulations offers a cost-effective approach by reducing the cost of concentrate mixture (INR 2210/tonne). Furthermore, the comparable growth performance, feed utilization efficiency, and health status observed in the present study indicate that this economic advantage can be achieved without compromising animal productivity or well-being. Therefore, the use of guar korma as a partial replacement for soybean meal can economically benefit farmers, particularly those operating under smallholder farming systems. Since, compositional characterization of anti-nutritional compounds and processing-related changes of roasted guar korma was not evaluated in the present study, the observed responses of animal performances cannot be conclusively attributed to the roasting process itself. Therefore, further investigations are required to determine whether these responses resulted from the effects of roasting or from the reduction of anti-nutritional activity. In addition, future research directions could be broadened beyond lactating animals to include higher inclusion levels, long-term feeding studies, and a more detailed assessment of nutrient utilization and metabolic responses.

## Data Availability

The original contributions presented in the study are included in the article/supplementary material, further inquiries can be directed to the corresponding author.

## References

[1] Mitra A Tripathy AK Singh RK Bujarbaruah KM. "Transforming the Indian livestock sector". In: Bansal KC Lakra WS Pathak H, editors. Transformation of Agri-Food Systems. Singapore: Springer (2023)

[2] DAHD. 20th Livestock Census-2019 All India Report. New Delhi, India: Ministry of Fisheries, Animal Husbandry and Dairying, Department of Animal Husbandry and Dairying, Government of India (2019). p. 1–42.

[3] Chiariotti A Borghese A Boselli C Barile VL. Water buffalo’s adaptability to different environments and farming systems: a review. Animals. (2025) 15:1538. doi: 10.3390/ani15111538, 40509004 PMC12153804

[4] Malenica D Kass M Bhat R. Sustainable management and valorization of agri-food industrial wastes and by-products as animal feed: for ruminants, non-ruminants and as poultry feed. Sustainability. (2022) 15:117. doi: 10.3390/su15010117

[5] Makarabbi G Sabu A Saxena N Sharma ML. An analysis of knowledge, attitude and practice gaps in scientific buffalo husbandry. PLoS One. (2025) 20:e0327208. doi: 10.1371/journal.pone.0327208, 40743269 PMC12312951

[6] Vithalrao US Chandrakar P Mahanthesh MT Singh G Sudhakar S Tanpure MU . Advances in nutritional strategies for enhancing livestock productivity: a review. Arch Curr Res Int. (2025) 25:138–56. doi: 10.9734/acri/2025/v25i121658

[7] Tripathi SK Randhawa GS. "Guar: an industrial crop from marginal farms". In: Bhullar GS, Bhullar NK, editors. Agricultural Sustainability: Progress and Prospects in Crop Research. Waltham, USA: Academic Press (Elsevier Inc.) (2012). p. 47.

[8] APEDA. Dept. of Commerce, Ministry of Commerce and Industry, Govt. of India. (2025). Deptt. of Commerce. Available online at: https://apeda.gov.in/apeda-product-catalogue (Accessed April 20, 2026).

[9] Shrestha R. Studies on agro-ecological performance and crop physiology of guar. (doctoral dissertation). The Graduate and Professional School of Texas A&M University, USA, (2022).

[10] Lee J Connor-Appleton A Haq CB Bailey A Cartwright A. Quantitative measurement of negligible trypsin inhibitor activity and nutrient analysis of guar meal fractions. J Agric Food Chem. (2004) 52:6492–5. doi: 10.1021/jf049674+, 15479012

[11] Conner SR Lee JT Carey J Bailey CA. Nutrient characterization of guar meal fractions. Poult Sci. (2001) 80:50.

[12] Nidhina N Muthukumar SP. Antinutritional factors and functionality of protein-rich fractions of industrial guar meal as affected by heat processing. Food Chem. (2015) 173:920–6. doi: 10.1016/j.foodchem.2014.10.071, 25466107

[13] Abdel-Wahab W Sayed SK Sabek RA Abbas MS Sobhy HM. Effect of using guar korma meal as a new source of protein on productive performance of buffalos. Asian J Anim Sci. (2016) 10:300–6.

[14] Shweta N Rani KS Kumar DS Illa SK. Guar meal as an alternative protein source: effects on digestibility and growth performance of Nagavali ram lambs. Trop Anim Health Prod. (2025) 57:430. doi: 10.1007/s11250-025-04685-0, 41055818

[15] Shwerab AM Khalel MS Khayyal AA Hassan AA Yacout MH. Influence of different treatments of guar korma meal on sheep performance. Egyptian J Nutr Feeds. (2015) 18:49–63.

[16] Janampet RS Malavath KK Neeradi R Chedurupalli S Thirunahari R. Effect of feeding guar meal on nutrient utilization and growth performance in Mahbubnagar local kids. Vet World. (2016) 9:1043–6. doi: 10.14202/vetworld.2016.1043-1046, 27847410 PMC5104709

[17] Sharif M Nazar M Sultan JI Bilal MQ Shahid M Hussain A. Effect of replacing cotton seed cake with guar meal on growth performance and economics in Sahiwal calves. J Anim Plant Sci. (2014) 24:28–32.

[18] AOAC. Association of Official Analytical Chemists. Official Methods of Analysis. 18th ed. Gaithersburg: AOAC International (2007).

[19] Van Soest P Robertson J Lewis B. Methods for dietary fiber, neutral detergent fiber, and nonstarch polysaccharides in relation to animal nutrition. J Dairy Sci. (1991) 74:3583–97. doi: 10.3168/jds.S0022-0302(91)78551-2, 1660498

[20] SPSS. Statistical Packages for Social Sciences. Version 30.0.0. IL: SPSS Inc (2024).

[21] Snedecor G Cochran W. Statistical Methods. 8th ed. New Delhi: East West Press Pvt. Ltd. (1994). p. 313.

[22] Goswami A Thakur SS Amrutkar SA. Growth and nutrient utilization in calves fed guar (*Cyamopsis tetragonoloba*) meal replacing ground nut cake in concentrate with and without added sweetner and flavour. Indian J Anim Nutr. (2012) 29:40–5.

[23] Jongwe C Thakur SS Kaur J Mahesh MS. Effect of replacing groundnut cake with guar meal (*Cyamopsis tetragonoloba*) in concentrate mixture with and without added sweetener and flavour on production performance of Sahiwal cows. Indian J Anim Nutr. (2014) 31:138–42.

[24] Biel W Jaroszewska A. Compositional and nutritional evaluation of guar (*Cyamopsis tetragonoloba* L.) meal. Anim Nutr Feed Technol. (2019) 19:385–93. doi: 10.5958/0974-181X.2019.00036.2

[25] Shekhawat S. S. Effect of dietary inclusion of rumen protected malic acid heat treated proteins on intake, nutrient utilization, methane emission, milk production and quality in early lactating buffaloes. (MVSc thesis), ICAR-National Dairy Research Institute (Deemed University), Karnal, Haryana, India (2025), pp. 1–107.

[26] Ojha BK Singh P Verma AK Patil AK. Effect of supplementation of de-oiled mahua seed cake and guar meal on the nutrient utilization and growth performance in crossbred calves. Indian J Anim Nutr. (2012) 29:222–5.

[27] Chhikara S Kishore N Dahiya SS Yadav DK Khaliya J Singh S. Growth performance and nutrient utilization in growing buffalo calves as affected by replacing groundnut cake with roasted guar korma (*Cyamopsis tetragonoloba*). J Entomol Zool Stud. (2020) 8:1248–1251. doi: 10.22271/j.ento

[28] Shweta N Rani KS Kumar DS Illa SK. Guar meal as a substitute for conventional soybean meal: impact on blood and serum profile of Nagavali ram lambs. Int J Bio-Resour Stress Manag. (2025) 16:01–8. doi: 10.23910/1.2025.6119

[29] Mani AMM. Potential impact of non-conventional protein resources *Cyamopsis tetragonoloba* (guar meal) in lamb’s diet. Acta Vet Eurasia. (2024) 50:173–9. doi: 10.5152/actavet.2024.24007

[30] Yadav DK Chhikara S Kumar G Gaur P Kansal G Kumar P. Groundnut cake was replaced with roasted guar (*Cyamopsis tetragonoloba*) to observe the effects on development and nutrient utilization in developing buffalo calves korma. Int J Adv Biochem Res. (2024) 8:886–9. doi: 10.33545/26174693.2024.v8.i1Sl.468

[31] Bargo F Reate DH Santini FJ Muller LD. Ruminal digestion by dairy cows grazing winter oats pasture supplemented with different levels and sources of protein. J Dairy Sci. (2001) 84:2260–72. doi: 10.3168/jds.S0022-0302(01)74673-5, 11699458

[32] Dey A Thakur S Singh RK Sheoran S Andonissamy J Kumar S. Developing a feeding module with a blend of garlic oil and cinnamon bark for enhancing antioxidant status and immunity of Murrah buffalo (*Bubalus bubalis*) with an improvement in feed efficiency and reduced methane emissions. Antioxidants (Basel). (2025) 14:702. doi: 10.3390/antiox14060702, 40563334 PMC12189884

[33] Dhillon KS Randhawa CS Gupta K Singh RS Chhabra S. Reference values for haematological and biochemical profile in adult Indian buffaloes. Buffalo Bull. (2020) 39:145–54. Available online at: doi: http://kuojs.lib.ku.ac.th/index.php/BufBu/article/view/2108

[34] Carr JH. Clinical Hematology Atlas-E-Book. Sixth ed. Canada: Elsevier Health Sciences (2021) (ISBN: 978-0-323-71192-0).

[35] Soliman MS El-Okazy AM Abu Hafsa SH. Effect of partially or totally replacing soybean meal by guar korma meal on sheep and cows performance milk production. J Anim Poult Prod. (2014) 5:43–55. doi: 10.21608/jappmu.2014.68607

[36] Thombare N Jha U Mishra S Siddiqui MZ. Guar gum as a promising starting material for diverse applications: a review. Int J Biol Macromol. (2016) 88:361–72. doi: 10.1016/j.ijbiomac.2016.04.001, 27044346

[37] NRC. National Research Council. Washington, DC: National Academics Press (2001).

